# Approach to Patellar Dislocation Treatment: Review and Prospects

**DOI:** 10.1111/os.70105

**Published:** 2025-09-05

**Authors:** Djovensky Gateau, Dereje Gobena Alemayehu, Zhi Zhang, Hanyu Wang, Bygrage Mayendesa, Elena Tahir, Xing Ma

**Affiliations:** ^1^ Orthopedic Surgery Department First Affiliated Hospital of Xi'an Jiaotong University Xi'an Shaanxi China

**Keywords:** biomechanical modeling, lateral patellar dislocation, MPFL reconstruction, risk stratification, tibial tubercle osteotomy, trochlear dysplasia

## Abstract

Lateral patellar dislocation (LPD) is a musculoskeletal condition characterized by a complex etiology. Despite significant advancements in management strategies, it continues to pose considerable challenges. Critical anatomic risk factors previously identified include trochlear dysplasia (TD), patella alta, and elevated tibial tubercle–trochlear groove (TT‐TG) distance, with TD being the most significant. A thorough risk assessment using predictive models is primarily recommended to assist in patient counseling and to identify high‐risk cases, for whom early surgical intervention may be considered. Controversies persist regarding the indications for combined surgical procedures, including tibial tubercle osteotomy (TTO), derotational distal femoral osteotomy (DDFO), and lateral retinacular release (LRR) with medial patellofemoral reconstruction (MPFLR). Moreover, emerging evidence suggests that a deeper understanding of the interplay between anatomic factors may optimize surgical prioritization and improve clinical outcomes. The combined surgical approach should be reserved for meticulously selected cases with substantial anatomic risk factors, while isolated MPFLR may prove adequate for cases with milder grade risk factors. To enhance individualized treatment strategies and improve outcomes for patients with LPD, deeper insights into the interaction of anatomical factors, supported by higher‐quality clinical research and advancements in biomechanical modeling, are essential.

## Graphical abstract

Lateral patellar dislocation treatment should be individualized by integrating risk stratification tools with assessment of anatomical risk factor complexity. A clinical decision flowchart is proposed to guide treatment strategy and highlight the importance of risk‐specific surgical planning.

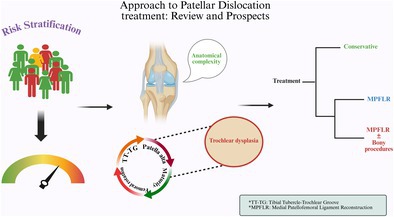

**Abbreviations**
LPD: lateral patellar dislocation
PF: patellofemoral
MPFLR: medial patellofemoral ligament reconstruction
FE: finite element
SA: sulcus angle
ISI: insall–salvati index
CDI: caton deschamps index
LRR: lateral retinacular release
TTO: tibial tubercle osteotomy
TP: trochleoplasty
FAA: femoral anteversion angle
DDFO: derotational distal femoral osteotomy
TD: trochlear dysplasia
TT‐TG: tibial tubercle‐trochlear groove
MR: magnetic resonance
CT: computer tomography
TT‐LTR: tibial tubercle to the lateral trochlear ridge
PT‐LTR: patellar tendon to the lateral trochlear ridge
PSR: patellar shift ratio

## 1 Introduction

Lateral patellar dislocation (LPD) is a musculoskeletal disorder predominantly impacting young and active individuals. An accurate diagnosis requires comprehensive knowledge of the various forms of LPD and the mechanisms leading to the injury.

In most instances, LPD occurs due to a non‐contact mechanism characterized by altered lower limb kinematics (e.g., knee valgus), especially in individuals with pre‐existing anatomical abnormalities [1].

Three types of LPD are described in the literature: acute (primary), recurrent, and fixed or habitual. The annual incidence of primary LPD is estimated at 42 per 100,000 person‐years [2]. Patients typically describe the injury as a sudden, non‐contact knee twisting, often accompanied by a distinct ‘pop ‘sound and a sensation of the knee giving way. Approximately one third of individuals with acute LPD will experience recurrent instability or redislocation [2, 3]. Patients with recurrent symptoms frequently report pain, clicking, and catching, particularly during activities involving knee flexion, such as stair climbing and squatting. These symptoms can significantly impair daily functioning and increase the risk of developing patellofemoral (PF) osteoarthritis. Fixed or habitual LPD is usually linked to soft tissue laxity or contracture, which may be associated with underlying genetic conditions. Thus, a thorough medical history should involve evaluating hereditary factors [4].

Clinicians may initially evaluate the type of dislocation and associated risk factors through a combination of physical tests. A positive patella apprehension test reveals observable discomfort or involuntary quadriceps contraction (guarding) during lateral displacement in patients with known PF instability. The patellar tilt test measures both lateral tilt and tightness of the lateral retinaculum (LR), and the J‐sign, which indicates a high‐grade dysplastic trochlea, may be observed during dynamic assessment of patellar tracking [5, 6]. Additionally, examining the extensor mechanism is crucial. Any quadriceps atrophy, especially of the vastus medialis obliquus (VMO), should be noted.

**FIGURE 1:**
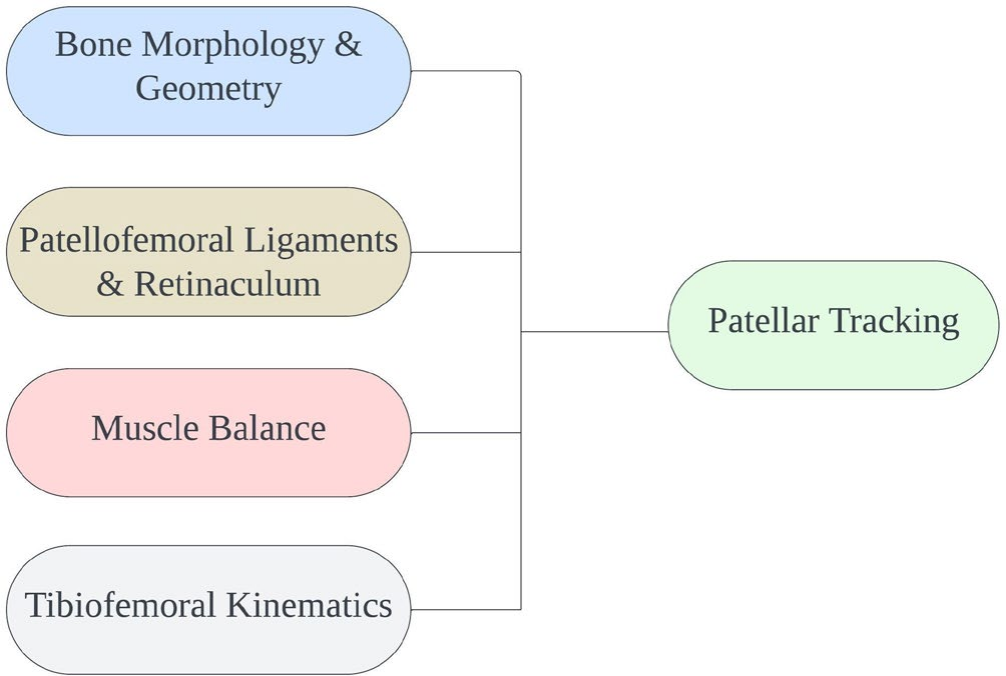
Key factors influencing patellar tracking.

A solid understanding of patellofemoral biomechanics is essential for guiding appropriate interventions (Figure 1). Considering the complex interactions between soft tissues and bone structure, several anatomical risk factors for LPD have been identified. These include trochlear dysplasia (TD), a high‐riding patella (patella alta), and an increased tibial tubercle–trochlear groove (TT‐TG) distance. Recognizing these anatomical contributors is essential for assessing the risk of recurrence and planning treatment.

To complement clinical findings, imaging using anteroposterior, lateral, and skyline radiographs, magnetic resonance imaging (MRI), and computed tomography (CT) is essential for identifying concomitant injuries (e.g., osteochondral fractures) and confirming the risk factors present. MRI efficiently assesses the integrity of the medial patellofemoral ligament (MPFL) and any associated ligamentous damage. A CT scan of both lower limbs is utilized to evaluate coronal alignment.

Key radiological parameters used in LPD assessment include the TT–TG distance, Dejour's trochlear classification, and the femoral sulcus angle (SA), which enable qualitative and quantitative evaluations of trochlear morphology [7]. Patella height is assessed using indices such as the Insall–Salvati (ISI) and Caton–Deschamps (CDI). Additionally, PF and patella tilt angles contribute to quantifying the patella's lateral inclination.

In the absence of significant osteochondral damage, conservative treatment is typically recommended for acute LPD. However, the high recurrence rate and functional decline associated with non‐operative management suggest that surgical intervention may be more suitable in some cases.

Given the individual variation in clinical presentation, classification systems are essential for informing treatment decisions. Patients presenting with LPD may be classified utilizing the WARPS/STAID classification system [8]. Predictive measures such as patellar scores have also been proposed for determining individual recurrence risk [9, 10].

Multiple surgical interventions can be performed, including medial patellofemoral ligament reconstruction (MPFLR), tibial tubercle osteotomy (TTO), and trochleoplasty (TP). Nonetheless, there are ongoing debates regarding the indications for combining some procedures.

This review presents the predictive methods proposed for anticipating recurrent LPD and the current evidence on surgical treatment strategies, particularly emphasizing the combined surgical approach. Furthermore, the potential to improve the accuracy of predictive models and clinical outcomes in managing LPD is discussed.

## 2 Literature Screening and Selection

**FIGURE 2:**
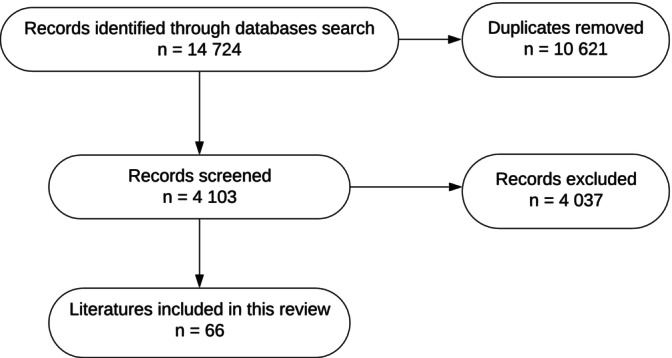
Flowchart literature screening and selection.

Databases including PubMed, Web of Science, and Scopus were comprehensively screened to identify studies discussing the epidemiology, treatment, and recurrent risk of LPD (Figure 2). Search terms included: “lateral patellar dislocation,” “recurrent lateral patellar dislocation,” “lateral patellar dislocation prediction,” “lateral patellar dislocation risk,” and “patellofemoral instability treatment.”

Boolean operator AND was used to combine “MPFLR” with the following surgical procedures to identify relevant studies discussing surgical combinations: MPFLR AND TTO, MPFLR AND lateral release, MPFLR AND trochleoplasty, and MPFLR AND derotational distal femoral osteotomy (DDFO). The search was limited to English‐language articles published between 2000 and 2024.

Peer‐reviewed original research articles, narrative reviews, systematic reviews, meta‐analyses, and biomechanical studies relevant to LPD treatment were included in the final review. Articles were excluded if they had irrelevant research topics, were unavailable in full text, involved animal studies, or were case reports. Multiple similar articles were also excluded to minimize redundancy and ensure that the final synthesis represented the most relevant and highest‐quality evidence available.

Ultimately, the final review included 66 studies: 22 systematic reviews and meta‐analyses, one randomized controlled trial, six prospective cohort studies, 27 retrospective cohort studies, four biomechanical studies, three narrative reviews, and three cross‐sectional studies.

## 3 Predictive Methods

### 3.1 Development of Predictive Models

The medial patellofemoral ligament (MPFL) is essential in counteracting the patella's lateral shift during initial knee flexion [11]. With increased flexion, the stability of the patella depends primarily on the morphology of the trochlear groove. In a dysplastic trochlea, there is insufficient containment of the lateral force exerted on the patella, which increases the risk of dislocation. At the same time, a high‐riding patella (patella alta) delays engagement with the groove, thereby increasing lateral patellar displacement.

Another important anatomical factor is the alignment of the tibial tubercle. An excessively lateralized position relative to the trochlear groove also lateralizes the patellar tendon's force vector, increasing the risk of instability.

The likelihood of LPD increases significantly when multiple abnormalities coexist [12]. Some anatomical features may have limited influence when isolated, but become critical when combined with more dominant factors. For example, the effect of a mildly elevated TT–TG distance may be minimal in isolation but problematic when paired with TD or patella alta.

Using advanced statistical methods, researchers assessed the predictive value of each risk factor to gain a greater understanding of these interconnected variables. The findings have established the groundwork for developing scoring systems that facilitate risk stratification.

#### 3.1.1 Predictive Models/Scores

Several models have been developed to quantify the risk of recurrent LPD by integrating key anatomical and demographic risk factors.

Balcarek et al. [9] introduced the Patellar Instability Severity (PIS) score, which includes patient age, TD grade, and patella alta. Although patella alta, TT–TG distance, and bilateral instability were not statistically significant predictors, they were included in the PIS score due to their high odds ratios. Patients scoring ≥ 4 out of 7 were deemed at high‐risk recurrence within 2 years.

Building on this, Jaquith et al. [13] retrospectively evaluated 222 patients (≤ 18 years) and identified four key predictive factors: TD (Dejour A–D), skeletal immaturity, contralateral dislocation, and CDI > 1.45. The presence of all four factors predicted an 88% risk of recurrence within 1 year, which decreased with fewer factors present: 75% with three, about 55% with two, 30% with one, and 14% with none. Their findings aligned with previous finite element (FE) models [12], demonstrating risk amplification when multiple abnormalities were present.

To further expand on this concept, Arendt et al. [14] prospectively followed 145 patients with acute LPD over 2 years. The top predictors for recurrence were TD and skeletal immaturity, followed by patella alta (ISI ≥ 1.3). This model estimated a 79% recurrence risk with all three factors present, declining to 51%, 23%, and 5.8% with two, one, or no factors, respectively.

In 2019, Hevesi et al. [10] proposed the Recurrent Instability of the Patella (RIP) score, which is based solely on factors deemed significant in multivariate analysis, including TD, patient age < 25, skeletal immaturity, and TT–TG/patellar ligament (PL) ratio. With a total of 5 points, the RIP score stratifies patients into low (0–1), intermediate (2–3), and high‐risk (4–5) categories. It demonstrated 89.7% specificity for high‐risk patients and 100% sensitivity for low‐risk cases over a 10‐year follow‐up.

Wierer et al. [15] later developed the Patellar Instability Probability (PIP) calculator, incorporating only age (≤ 16), bilateral LPD, and TD. At the same time, they proposed a modified PIS (mPIS) score, which excludes patella alta, TT–TG distance, and patellar tilt. The PIP predicted recurrence within 2 years with 79% accuracy, outperforming the mPIS (76%) and original PIS (66%).

In contrast, Yu et al. [16] reported higher predictive and negative values for the PIS score at 92.9% and 85.1%, respectively. Of 171 recurrent LPD patients, PIS was validated in 143, compared to 96 for RIP and 83 for PIP. This suggests that even statistically less significant factors, such as patella alta and tilt included in the PIS score, hold clinical importance in predicting LPD.

#### 3.1.2 Evaluation of Key Risk Factors

Across these models, some variables show more consistent predictive value than others. TD and skeletal immaturity repeatedly emerged as the most significant predictors. However, there is notable disagreement regarding the overall influence of patella alta, TT–TG distance, and patellar tilt.

##### 3.1.2.1 Trochlear Dysplasia (TD)

Although TD predominantly appeared as a primary predictor across models, its evaluation methods vary. Some researchers rely on the qualitative Dejour classification. In contrast, others, like Wierer et al. and Arendt et al., used the lateral trochlear inclination (LTI) and the femoral sulcus angle (SA ≥ 154°), respectively. While subjective methods remain in use, quantitative metrics like LTI could facilitate correlation with TD grades [17].

Furthermore, in models such as the PIS, TD grades are differentiated by assigning different scores (0–2 points) based on severity. On the other hand, other researchers did not distinguish between Dejour types A through D.

##### 3.1.2.2 Patella Alta

Cut‐off values for defining patella alta differ among indices, with thresholds ranging from 1.2 to 1.45. Arendt et al. concluded that patella alta was a significant predictor when using ISI (≥ 1.3) but not CDI, while Wierer et al. found no predictive significance with either method. This inconsistency may stem from methodological differences or population variability. Moreover, these discrepancies suggest that employing a range of severity grades (e.g., mild, moderate, severe patella alta) could yield a more accurate assessment than relying on a fixed cut‐off value.

##### 3.1.2.3 TT‐TG Distance

Building on the concept of PF individual characteristics, Hevesi et al. favored the TT–TG/PL ratio due to its greater diagnostic sensitivity compared to the TT‐TG distance. Nonetheless, Wierer et al. reported no significant predictive power for either parameter, highlighting the dependency of the predictive performance of such parameters on how they interact with more critical anatomical factors, such as TD.

##### 3.1.2.4 Age vs. Skeletal Maturity

While the PIS score considers age alone, the RIP score specifically factors in skeletal immaturity alongside age, acknowledging differences in maturation timing. Jaquith and Arendt et al. also found that skeletal immaturity was a stronger predictor than age, highlighting the significance of individual growth assessments in risk evaluation.

##### 3.1.2.5 Bilateral Involvement

Only the PIS and PIP scores include bilateral LPD as a predictor. Arendt et al. assessed contralateral dislocation but found no predictive value, while Jaquith et al. reported it as significant. This may have reflected the younger demographic in Jaquith's study, consistent with previous findings showing a higher bilateral LPD incidence in patients < 18 years [18].

### 3.2 Extensor Mechanism Containment

The PF containment refers to the axial alignment of the PF joint. This term has recently emerged in discussions regarding PF instability. The assessment of PF containment has expanded beyond the traditional TT–TG distance to incorporate various axial MRI parameters. These include the patellar tendon to the lateral trochlear ridge (PT‐LTR) distance and the tibial tubercle to the lateral trochlear ridge (TT‐LTR) distance [19, 20].

The PT–LTR distance measures the patellar tendon's width lateral to the trochlea. A distance exceeding 9 mm is linked to a higher risk of recurrent LPD, while a distance of 2 mm suggests a lower risk. However, the assessment of lateral displacement must be integrated with other factors for a comprehensive risk assessment. Thus, similarly to other parameters, the PT–LTR measurement should not be evaluated in isolation.

One of the most recent axial parameters suggested is the TT–LTR distance, which reflects the position of the tibial tubercle relative to the trochlea. A positive TT–LTR value indicates lateralisation of the tibial tubercle, while a negative value suggests a more medial position, potentially reducing the risk of lateral instability. Weltsch et al. [19] have shown that in adolescents, the TT–LTR distance was the only parameter among the new axial parameters to predict recurrent LPD independently. A threshold of > −1 mm was identified as a critical value.

The patellar shift ratio (PSR), introduced by Chen et al. [21], presents another innovative measure of PF containment. The PSR calculates the degree of lateral patellar displacement relative to the trochlea, and a threshold of 24.3% is associated with a higher risk of recurrent LPD. The PSR showed impressive diagnostic capabilities with a sensitivity rate of 96.3% and a specificity rate of 85.2%. While the PSR shows promising results, it should be noted that this measurement depends on patellar glide, which can be affected by different biomechanical factors. Thus, the PSR requires evaluation together with other radiological data to achieve a complete risk assessment.

### 3.3 Limitations and Clinical Significance

**FIGURE 3:**
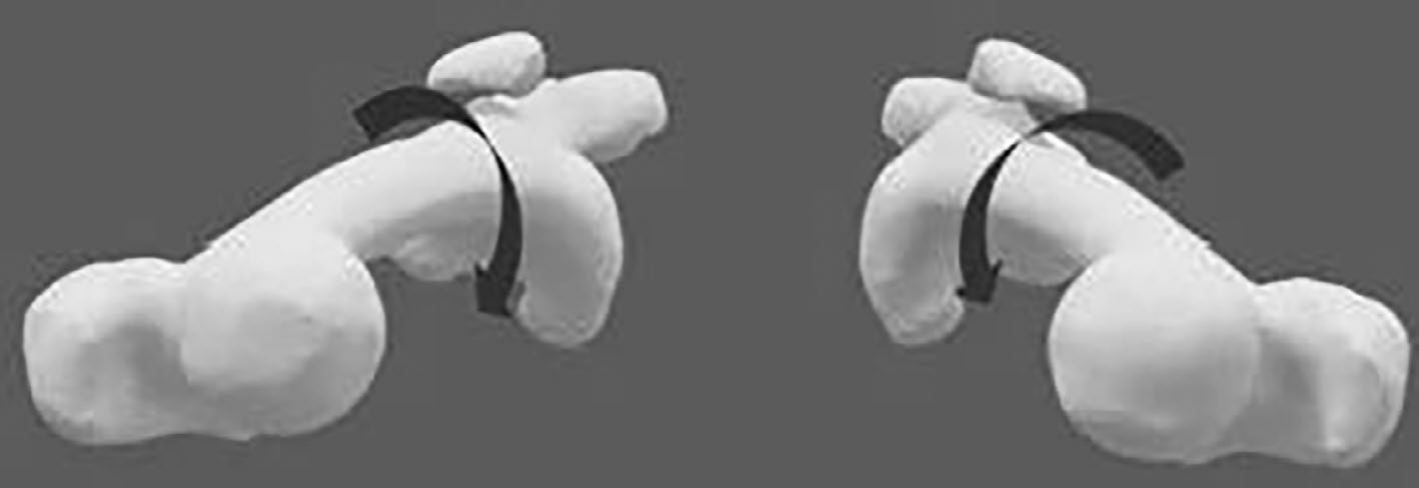
3D reconstruction of a patient's lower limbs (top view) with bilateral recurrent lateral patellar dislocation. Arrows indicate femoral anteversion contributing to structural internal femoral rotation. Additional identified risk factors include trochlear dysplasia and increased TT–TG distance.

The discrepancy between variable inclusion and measurement standards hinders cross‐comparison between the predictive models. Notably, none of the models account for rotational limb deformities, such as femoral anteversion (Figure 3), which are increasingly recognized as significant contributors to PF instability [22]. The variation in risk factors assessment methods highlights the necessity for standardized evaluation methodologies. Without such consensus, the accuracy of scoring systems may diminish, particularly for borderline cases.

Despite these limitations, all major predictive models recognize TD, skeletal immaturity, and age as critical risk factors for recurrent LPD. The addition or exclusion of secondary factors such as patella alta, tilt, and TT–TG distance depended on study designs and cohorts; therefore, using a score that includes most key parameters is preferable for generality. In clinical practice, these tools (Tables 1 and 2) can provide substantial assistance, particularly in counseling patients and identifying high‐risk patients who may require early surgical stabilization. However, larger‐scale studies with standardized protocols are essential for validating the accuracy of these predictive models.

**TABLE 1:** The risk factors and their evaluation methods are shown for each predictive model.

Risk factors	Predictive models	
	Jaquith et al. [13]	Arendt et al. [14]
Dejour A—D	1	
Femoral SA ≥ 154°		1
Skeletal Immaturity	1	1
Bilateral Instability	1	
CDI ≥ 1.45	1	
ISI ≥ 1.3		1
Score = Recurrent LPD Risk Percentage	4 = 88.4%, 3 = 69.2%–79.5%, 2 = 42.4%–63.5%, 1 = 24.8%–36.4%, 0 = 13.8%	3 = 78.5%, 2 = 43%–55%, 1 = 16%–23%, 0 = 5.8%

*Note:* Each factor is assigned 1 point depending on its presence or absence. If one to three factors are present, the percentage risk is determined based on the order of significance.

**TABLE 2:** The evaluation methods and scores attributed to each risk factor are shown for each predictive score.

Risk factors	Predictive Scores			
	PIS	mPIS	RIP	PIP
Age (years)				✔
< 25			2	
> 16	0	0		
≤ 16	1	3		
Skeletal Immaturity			1	
Bilateral Instability				
No	0	0		0
Yes	1	1		1
Trochlear dysplasia				
None	0	0		
Mild	1	1		
Severe	2	2		
Dejour A – D			1	
LTI				✔
Patellar height				
≤ 1.2	0			
> 1.2	1			
TT‐TG (mm)				
< 16	0			
≥ 16	1			
TT‐TG/PL ≥ 0.5			1	
Patellar Tilt* (°)				
≤ 20	0			
> 20	1			
Total Points	0–3 = Low risk	0–2 = low risk	0–1 = Low risk	*PIP = 1/(1 + EXP(−PIOR)). *PIOR = 3.5 + Age(years) × (−0.1) + contralateral instability × 0.7 + LTI × (−0.1)
	4–7 = High risk	3–6 = High risk	2–3 = Intermediate risk	
			4–5 = High risk	

*Note:* The PIP score is calculated using the patient’s age and the measured LTI angle in the formula*.

The newly developed axial parameters, such as PT–LTR, TT–LTR, and PSR, improve our ability to evaluate PF containment beyond the conventional TT–TG distance. Still, a single measure should not be used in isolation since risk assessment in PF instability requires a whole framework in which these measurements could be included. Their predictive effectiveness must be validated with further studies to enhance their practical application.

## 4 Treatment Approach

LPD treatment primarily aims to prevent recurrent dislocation and enhance knee functionality. Depending on concomitant injuries and the patient's risk of recurrence, this can be achieved through conservative or operative intervention.

Conservative treatment is generally recommended for patients diagnosed with acute LPD without additional injuries. This approach involves a brief period of immobilization using braces, casts, or splints, followed by a structured outpatient physiotherapy program to improve muscular balance and range of motion.

Although conservative treatment has shown effectiveness, especially in those at low risk for recurrence, the evidence supporting it is of limited quality. Flores et al. [23] reported a notable inconsistency in rehabilitation recommendations within the literature, particularly concerning the type, frequency, and structure of prescribed exercises. Furthermore, long‐term follow‐up data are scarce for patients who have received non‐operative treatment.

For instance, Magnussen et al. [24] found that among 104 patients managed conservatively after a primary LPD episode, 73% did not experience recurrence. However, only 26.4% could return to their desired level of sports participation without limitations at the three‐year mark. Recurrence rates reported for conservatively managed LPD patients vary significantly, ranging from 17% to 33% [2, 3], prompting a reevaluation of the universal application of conservative strategies in uncomplicated cases.

Surgical intervention is typically recommended when imaging indicates associated injuries, such as osteochondral fractures, or in recurrent cases. Procedures may include arthroscopic debridement, fixation of osteochondral fragments, or MPFLR with or without bony procedures.

While surgery leads to lower redislocation rates, its advantages regarding functional outcomes are still unclear. A meta‐analysis [25] concluded that the long‐term differences in redislocation and functional scores were not statistically significant after surgery compared to conservative treatment. However, clinical outcomes improved with the surgical approach up to 6 years. Another study involving children and adolescents showed that conservative treatment resulted in better pain outcomes, although the groups' redislocation rates or functional scores did not significantly vary [26].

However, it must be noted that many studies in published systematic reviews on surgical procedures did not specifically address anatomical factors, including high‐grade TD, severe patella alta, or increased TT–TG distance, which could have influenced their results. Recently, Liebensteiner et al. [27] launched the first randomized controlled trial using a customized surgical method in line with the individualized menu à la carte. This method could effectively address the demand for personalized treatment based on each patient's unique anatomical risk factors.

### 4.1 Surgical Procedures

Previously, LPD surgery focused on reestablishing patellar medial restraint through MPFL repair or MPFLR. It has evolved into a “menu à la carte” approach, allowing stabilization procedures to be performed in isolation or combined to address specific risk factors [6]. Beyond general complications such as infection and arthrofibrosis, each PF stabilization procedure has unique risks, as reported by Tompkins et al. [28]. Therefore, surgeons should balance the potential risk of complications and the benefits of stabilization procedures, particularly when considering combined methods.

#### 4.1.1 MPFLR

Due to its crucial role in lateral patellar restraint, MPFLR has become the systematic stabilization procedure in LPD patients with evidence of MPFL insufficiency. Physically active patients who undergo MPFLR can anticipate returning to their physical activities within 3 years [29]. However, a complication rate between 10.4% and 26%, including residual instability, revision surgery, and redislocation, is reported [30, 31]. No gold‐standard technique for MPFLR exists; however, success is optimized when the chosen method focuses on the anatomical placement of the femoral tunnel and proper graft tension.

It is essential to note that MPFL rupture results from LPD rather than being a causative factor [6]. Therefore, other risk factors contributing to MPFLR failure should be considered prior to surgery. When indicated, a TTO medialization or anteromedialization (AMZ) can be combined with MPFLR to rectify an elevated TT–TG distance, while tibial tubercle distalization corrects severe patella alta. Alternative soft tissue procedures, such as the Roux‐Goldthwait, can be implemented in skeletally immature patients to prevent physis damage. TP can be added to deepen the trochlea groove, whereas derotational osteotomy addresses excessive femoral anteversion and external tibial torsion.

#### 4.1.2 Tibial Tubercle Osteotomy (TTO)

A TT–TG distance of ≥ 20 mm is typically the threshold for TTO medialization. However, in studies investigating the benefits of combining TTO and MPFLR, indications range from 15 to 20 mm. While this procedure has gained popularity, research has reported conflicting results regarding its additional benefits when combined with MPFLR.

For instance, Ryan et al. [32] reported a greater return‐to‐sport rate and a reduced revision rate after 2 years for patients who had TTO medialization combined with MPFLR, compared to those who only had MPFLR. Conversely, Li et al. [33] found no notable difference in return to sport rates between these groups after 4 years.

Over the past decade, systematic reviews have highlighted this inconsistency. An analysis of 31 studies on TTO revealed no significant differences in redislocation rates or functional knee scores between patients with TT‐TG distances > 15 mm who received isolated MPFLR and those who underwent combined TTO medialization with MPFLR [34]. However, the combined group had a higher rate of return to sports.

Similarly, Guevel et al. [35] observed a low and comparable rate of recurrent instability following both interventions; however, patients who underwent isolated MPFLR had a better Kujala score.

One complicating factor in the studies is the variability in outcome measures. While most authors reported clinical outcomes such as recurrence rate, revision surgery, and functional knee scores, others highlighted the increased risk of postoperative complications of TTO, including the need for hardware removal.

Furthermore, the reporting on patient selection criteria is not comprehensive. Not all studies considered the overall impact of other anatomic risk factors, such as TD and patella alta, in selected patients for TTO.

Considering additional factors such as TD, Meng et al. [36] concluded that combining TTO and MPFLR leads to outcomes similar to those of isolated MPFLR without increasing postoperative complication rates. While the additional step of hardware removal following TTO was acknowledged, the authors emphasized that this combination may be more beneficial for patients with severe trochlear dysplasia and abnormal patellar tracking.

Another potential source of bias is the surgical approach reported. In some studies, TTO involved medialization and distalization of the tibial tubercle for patients with CDI ≥ 1.2, which may have potentially confounded outcomes. For instance, Zhou et al. [37] compared clinical outcomes between patients undergoing TTO medialization with MPFLR and TTO medialization + distalization with MPFLR. They reported no significant difference in clinical outcomes between the two groups. Similarly, a recent systematic review suggested that TTO distalization is unnecessary in cases with patella alta and increased TT‐TG distance. Although patella alta and increased TT‐TG distance have the same ultimate effect, their contributions to LPD pathomechanism are different and should ideally be investigated separately.

Interestingly, reports have suggested that non‐distalization procedures such as MPFLR and TTO medialization may decrease the patella height. Although MPFLR may reduce patellar height, this may not improve clinical outcomes according to Hiemstra et al. In contrast, Shih et al. observed a significant improvement in the Kujala score with reduced patella alta in patients who had an isolated MPFLR [38, 39]. Expanding on this debate, MPFLR, TTO medialization, and TP combined with MPFLR, also demonstrated a significant postoperative decrease in patellar height. However, this effect may only be seen in severe grade patella alta, since a study reported no significant differences in patella height in patients with preoperative mild patella alta following MPFLR and Insall proximal alignment [40, 41].

##### 4.1.2.1 Clinical Implications

The reports on the benefits of TTO combined with MPFLR notably vary in study designs. These limitations may have concealed the patient subgroups that could demonstrate the advantages of this approach. Identifying the appropriate patient subgroups and standardizing outcome measurements requires further investigation. Consequently, carefully considering patient characteristics is necessary when combining TTO with MPFLR.

When evaluating the tibial tubercle lateralisation, clinicians should consider the limitations of the TT‐TG distance as a predictor of recurrent dislocation and a parameter for assessing lateral patellar tracking. Incorporating individual characteristics such as the TT‐TG/PL distance and determining whether limb malrotation affects the TT‐TG distance measurement may refine patient selection.

Ultimately, combining TTO and MPFLR should be individualized by considering factors such as the degree of TT‐TG distance elevation and accompanying anatomical factors, particularly pathological trochlear morphology. In carefully selected patients, especially those with elevated TT‐TG distance > 20 mm, higher‐grade TD with significant evidence of patellar maltracking, TTO may enhance PF stability and improve functional prognosis without introducing a greater risk of postoperative complications. On the other hand, isolated MPFLR may suffice for patients with borderline elevated TT‐TG distance, mild TD, and no signs of maltracking. This approach eliminates the need for subsequent hardware removal procedures, reduces surgical complexity, and shortens the rehabilitation period.

In patients with clinically significant patella alta, TTO distalization is most effective in restoring normal patellar height. However, isolated MPFLR may suffice in some cases. For cases with mild patella alta, TTO distalization can be avoided, especially since there is potential reduction in patellar height with non‐distalization procedures. This approach would facilitate quicker recovery and prevent related complications such as tibial fracture and increased PF joint contact pressure due to excessive distalization of the tibial tubercle [42].

#### 4.1.3 Trochleoplasty (TP)

TP is highly effective in enhancing bony restraint on the patella and is generally indicated for severe grade TD (Dejour types B and D), particularly with a supratrochlear spur of > 5 to 7 mm or the presence of the J‐sign.

Some authors reported that TP performed in isolation or with other procedures yields improved knee functional score and a low recurrence rate. In contrast, others encourage combining TP with MPFLR due to the higher failure rate with isolated TP [43, 44]. However, acceptable clinical outcomes were also reported in patients with high‐grade TD treated with isolated MPFLR [45]. To provide further insights, Fitzpatrick et al. [46] utilized FE model analysis to demonstrate that combining TP with MPFLR conferred greater PF joint stability than isolated TP.

Nonetheless, the indication for TP remains unclear due to the heterogeneity in studies regarding TD assessment and outcome measures. At the same time, most studies did not comprehensively define the potential associated complications. A meta‐analysis of 1000 trochleoplasties performed in isolation and combined with other procedures revealed complications such as residual instability, the need for additional surgeries, and PF osteoarthritis [47].

Although TD and LPD are strongly associated, compared with other procedures, TP is not widely performed [44, 48]. This may reflect the lack of standardization in TD assessment and grading, making treatment guidance unclear for most surgeons. Furthermore, concern regarding growth plate damage in skeletally immature patients persists. Although favorable outcomes and no signs of cartilage degeneration were reported in adolescents with less than 2 years of growth, further validation studies are necessary before widespread adoption of TP in this population [49].

##### 4.1.3.1 Clinical Implications

Whether performed in isolation or combined with other procedures, TP offers a highly effective means to restore PF joint congruence and normal patellar tracking, thereby decreasing the recurrent dislocation rate. Additionally, biomechanical reports support clinical studies that suggest the combination of TP and MPFLR for a better prognosis.

Nevertheless, TP is a technically demanding procedure with risks of arthrofibrosis, PF osteoarthritis, and revision if not performed meticulously. Consequently, its combination with MPFLR should be carefully considered in appropriately selected patients while balancing TD severity, patients' demographic factors, and surgical expertise. Ultimately, the low quality of available studies warrants the need for randomized controlled trials to clarify TP indications in the LPD treatment algorithm.

#### 4.1.4 Derotational Osteotomy

Femoral anteversion angle (FAA), TD, and patellar tilt are strongly correlated, highlighting the impact of limb malrotation on patellar tracking [50, 51]. Interestingly, several studies have reported a notable improvement in the patellar tilt angle and a reduction in the TT–TG distance following DDFO [52, 53]. Thus, the evaluation of coronal limb alignment should be considered in patients who present these anatomical risk factors. Although Barton et al. suggested a threshold of > 25° for FAA and > 30° for external tibial rotation angle, the indication for DDFO varies among studies and generally ranges from 20° to 30° [22, 54].

Femoral and tibial derotational osteotomies have shown substantial efficacy in addressing limb malrotation in patients with PF instability, even in patients with high‐grade TD [55, 56, 57]. Furthermore, compared to isolated MPFLR, combining DDFO and MPFLR appears to offer greater improvements in functional knee scores with a relatively low complication rate. Additionally, it improves radiographic parameters such as TT‐TG distance and patellar tilt.

In a retrospective study, Hao et al. [58] reported that no redislocation cases or major complications were observed when comparing clinical outcomes in patients who underwent isolated MPFLR and DDFO combined with MPFLR. Patients treated with DDFO achieved better postoperative knee function scores and significantly improved TT–TG distance. Building on these findings, Huo et al. [53] compared clinical and radiological outcomes between patients undergoing TTO medialisation + MPFLR and DDFO + MPFLR. Both methods yielded a low recurrence rate, improved TT–TG distance, and restored normal patellar height (CDI < 1.2). However, regarding knee functional scores, DDFO achieved a more favorable outcome.

Recently published systematic reviews support these findings, suggesting that combining DDFO with MPFLR may yield greater functional knee scores while improving radiographic parameters in selected patients [22, 59, 60]. However, it must be noted that most studies are retrospective and employ different methodologies that may have introduced potential bias. Furthermore, the authors have no explicit agreement regarding when to combine DDFO and MPFLR.

There are four main opposing viewpoints on the indications for DDFO. While some authors first relied on a fixed cut‐off value of 20° to determine increased FAA, others considered a range of 20° to 30° to determine the high‐risk group for whom surgical intervention is indicated.

Another approach suggested is to perform DDFO in patients with an increased FAA associated with clinically evident patellar maltracking, shown with a high‐grade J‐sign. Finally, some authors argued that DDFO could be reserved for revision surgery following failure of soft tissue procedures in patients with increased FAA.

##### 4.1.4.1 Clinical Implications

Due to the weakness of available evidence, surgeons should resort to careful and individualized patient selection when considering the combination of DDFO with MPFLR. Moreover, derotational osteotomy is a technically demanding surgery carrying specific risks, such as hardware‐related complications, nonunion, and potential overcorrection that may lead to altered limb biomechanics.

DDFO can be considered in patients with a significant increase in FAA (25° and 30°), particularly those with clinical evidence of patellar maltracking, such as a high‐grade J‐sign. For cases with a mild increase in FAA ranging from 20° to less than 25° (20° ≤ FAA < 25°), but patellar tracking remains acceptable in dynamic assessment, isolated MPFLR may suffice to reduce the risk of morbidity with bony procedures. Future prospective research is warranted to refine surgical indications.

#### 4.1.5 Lateral Retinaculum Release (LRR)

Lateral retinacular release is no longer recommended as an isolated stabilization procedure in LPD patients, as it is significantly associated with increased PF joint instability. Nonetheless, it remains an adjunctive measure that can be performed along with other soft tissue and bony procedures. General indications for LRR include physical evidence of a tight LR and radiographic findings of increased patellar tilt. However, reports on its additional benefits when combined with other procedures vary.

In a prospective cohort study, Wang et al. [61] reported significantly higher postoperative knee scores after 1 year in patients who underwent MPFLR combined with LRR than in those who underwent isolated MPFLR. These findings are consistent with a systematic review [62] that revealed a greater improvement in knee scores with the combination approach. In contrast, Waters et al. [63] found no significant difference in clinical improvement after 1 year between patients treated with combined MPFLR and LRR and those treated with isolated MPFLR.

The differences in study design, particularly the exclusion and inclusion criteria, and the method used to assess patellar tilt, may partly explain these discrepancies. While Waters et al. conducted a retrospective study with a relatively small cohort, LRR was performed only in patients with an increased patellar tilt confirmed clinically and arthroscopically. Conversely, using imaging, Wang et al. measured and reported similar patellar tilt angle in both groups, which may have influenced the outcomes since no clear indication for LRR was defined. Moreover, the risk of failure appears to be higher in the combination group, suggesting that LRR may be associated with further instability. Kheir et al. [64] employed an FE model to demonstrate that combining MPFLR with LRR resulted in a 20% increase in the lateral patellar shift, highlighting LR's role in lateral patellar stability.

While Su et al. [65] have considered broader anatomical factors in patients who underwent TTO combined with MPFLR and LRR, their findings suggest that factors beyond LR tension, including female gender and TD grade, may significantly influence functional outcomes and failure rates.

##### 4.1.5.1 Clinical Implication

In light of LR's contribution to patellar stability and the inconsistent evidence regarding the benefits of combining MPFLR with LRR, this procedure should not be performed routinely in LPD patients. Appropriate candidates for added LRR should demonstrate clear clinical evidence of tight LR and persistent patellar tilt on imaging.

A thorough preoperative assessment, including dynamic patellar tracking evaluation and intraoperative examination, should complement imaging findings to further refine surgical indications. Furthermore, given the role of LR in PF stability as highlighted in biomechanical studies, the decision to add LRR must balance the goal of improving patellar tilt and LR tension against the risk of postoperative instability. LR lengthening may offer an alternative approach by preserving optimal lateral stability while addressing LR tension [66].

## 5 Prospects

**FIGURE 4:**
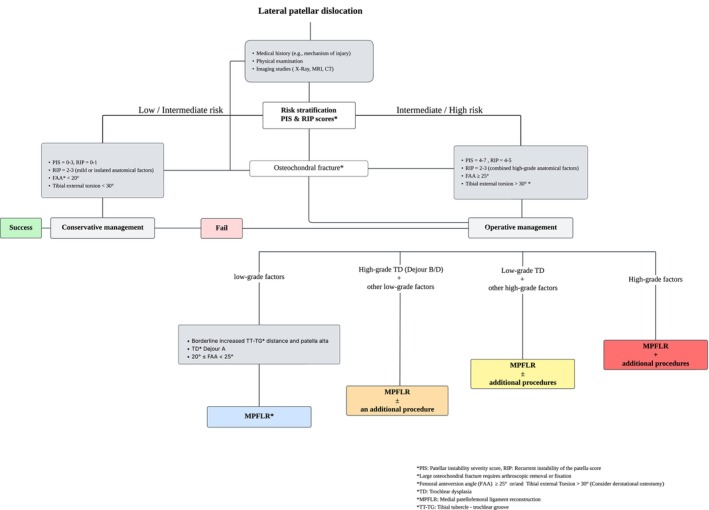
Following risk assessment, this flowchart delineates an evidence‐based approach to managing LPD based on the severity of anatomical factors. Integration of the PIS and RIP scores ensures a generalizable assessment, with TD as the primary risk factor and other features considered adjuncts. In cases of failed conservative treatment or greater anatomical complexity, management transitions from isolated MPFLR to combined procedures. The color‐coded boxes, from blue (isolated MPFLR) to red (combined procedures), reflect the intensity of the interventions.

Despite significant progress in treating LPD, notable persistent controversies in the assessment methods and indications for combined surgical procedures reflect the inherent complexity of this condition and the need for a comprehensive diagnostic workup aiming at individualized treatment (Figure 4). These challenges emphasize the need for reliable and validated clinical tools to identify patients at higher risk of LPD recurrence.

Orthopedic surgeons may mainly use the existing patellar predictive scores to advise their patients. Given the high recurrence rate following conservative treatment [2, 3], early surgical intervention may be considered in patients deemed high risk, particularly those presenting with three or more risk factors and young athletes. Conversely, patients categorized as low risk or those with minimal risk factors are most likely to achieve successful outcomes with non‐operative treatment.

Nevertheless, additional validation studies of the predictive scores are needed to enable a more comprehensive application in clinical decision‐making, particularly when evaluating borderline cases. Future studies should also focus on standardizing the assessment methods used in PF instability to refine these tools, enhance communication across studies, and improve guidance for the treating physicians. Moreover, clinical trials comparing conservative and operative treatment approaches may benefit from using the predictive scores as part of their inclusion and exclusion criteria [27].

TD, patella alta, and an elevated TT–TG distance are the predominant anatomical factors observed in patients with PF instability. However, when weighed independently, patella alta and an elevated TT–TG distance are not as strong predictors as TD; instead, they appear to magnify the risk when combined with a dysplastic trochlea, depending on their severity. Therefore, they may be regarded as adjunct risk factors.

The variation in cut‐off values for diagnosing patella alta and the potential variation in surgical outcomes across different grades of patella alta suggest that this condition may represent a more complex spectrum than currently understood. Consequently, categorizing patella alta cases into mild, moderate, or severe grades may offer greater insight into prognosis and treatment planning.

The “menu à la carte” approach promotes individualized treatment of patients with PF instability by correcting each significant anatomic factor present [6]. However, this strategy may not be feasible in some cases and carries increased surgical morbidity, longer rehabilitation periods, and considerable technical demands. Moreover, reports from the literature suggest that the benefits of combined procedures are not guaranteed.

Besides refining the indications for specific procedures, particularly osteotomies, understanding the underlying relationships between anatomical risk factors may be key to improving treatment strategies. The interplay between these factors could influence surgical decision‐making by promoting prioritization in correcting specific risk factors and performing fewer surgical interventions with potentially better outcomes.

For instance, the reported simultaneous improvement in TT‐TG distance, patellar tilt, and knee function scores following DDFO combined with MPFLR suggests that in cases presenting with increased FAA and TT‐TG distance, correcting femoral rotation may be prioritized over tibial tubercle medialization [22]. While TTO medialisation can improve the TT–TG distance, it does not address proximal malalignment. On the other hand, DDFO performed appropriately may concurrently optimize coronal limb alignment and improve TT–TG distance.

Another example is the potential decrease in patellar height observed following MPFLR, TTO medialisation, and TP. In patients with mild patella alta undergoing MPFLR and TTO, distalizing the tibial tubercle can be avoided if acceptable patellar height can be simultaneously achieved. Alternatively, if distalisation is warranted, the anticipated reduction in patellar height achieved with other procedures could be incorporated into surgical planning to minimize the risk of elevated PF contact pressure caused by excessive distalisation of the tibial tubercle [42]. Nevertheless, the complex relationships between patella alta, MPFL insufficiency, and elevated TT–TG distance need further clarification.

Overall, high‐quality prospective studies are essential to deepen the understanding of surgical indications for additional procedures. Parallel integration of patient‐specific FE models may complement clinical findings by clarifying the biomechanical effects of different surgical strategies.

## 6 Conclusion

Patellar predictive models provide clinicians with valuable prognostic tools to mainly inform patients on treatment expectations and assist in differentiating high‐risk cases, for whom early surgical treatment may be considered. Therefore, a comprehensive risk assessment should be integral to the initial clinical evaluation for LPD management.

Due to persistent controversies, combined surgical approaches should be reserved for carefully selected patients with substantial anatomic factors, while isolated MPFLR may suffice for cases with mild risk factors.

A deeper understanding of the complex relationship between anatomic factors, supported by higher‐quality clinical studies and advances in biomechanical modeling, is essential to refine individualized treatment strategies and optimize outcomes for patients with LPD.

## Author Contributions

**Djovensky Gateau:** conceptualization, writing – original draft, visualization. – **Dereje Gobena Alemayehu:** writing – review and editing. **Hanyu Wang:** writing – review and editing. **Bygrage Mayendesa:** writing – review and editing. **Elena Tahir:** writing – review and editing. **Zhi Zhang:** supervision, writing – Review and editing. **Xing Ma:** conceptualization, supervision, writing – review and editing.

## Conflicts of Interest

The authors declare no conflicts of interest.
